# Supplementary material for: C-Reactive Protein (CRP) Levels in Immune Checkpoint Inhibitor Response and Progression in Advanced Non-Small Cell Lung Cancer: A Bi-Center Study
Source: Cancers (Basel). 2020 Aug 17;12(8):2319. doi: 10.3390/cancers12082319 (PMC7464328; doi:10.3390/cancers12082319)
Supplement: Supplementary file 1 [file cancers-12-02319-s001.pdf]

Article

C-Reactive Protein (CRP) Levels in Immune Checkpoint Inhibitor Response and Progression in Advanced Non-Small Cell Lung Cancer: A Bi-Center Study

Jakob M. Riedl, Dominik A. Barth, Wolfgang M. Brueckl, Gloria Zeitler, Vasile Foris, Stefanie Mollnar, Michael Stotz, Christopher H. Rossmann, Angelika Terbuch, Marija Balic, Tobias Niedrist, Thomas Bertsch, Herbert Stoeger, Martin Pichler, Horst Olschewski, Gudrun Absenger, Joachim H. Ficker, Armin Gerger and Florian Posch

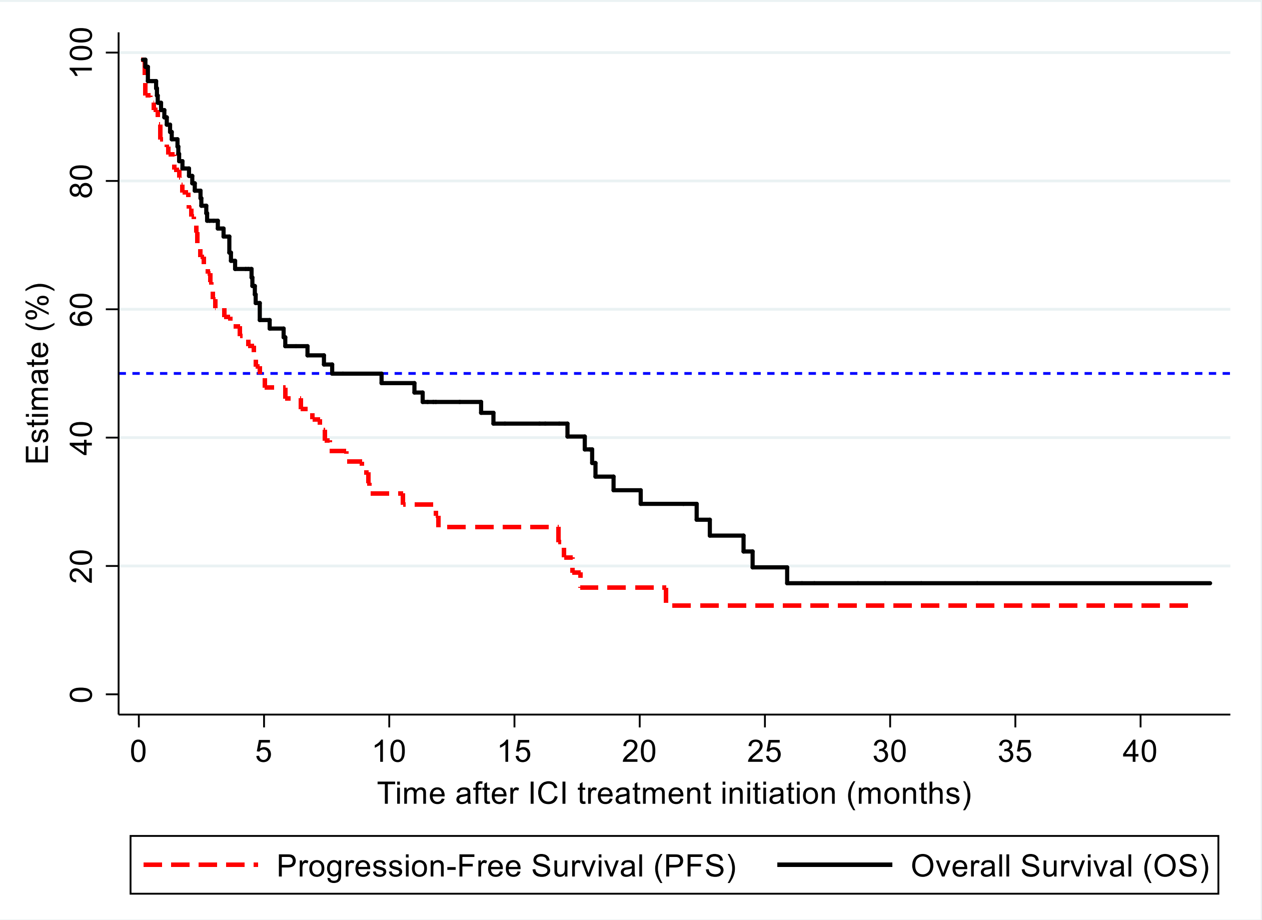

Figure S1. Progression-free and overall survival experience of the Graz cohort. Curves were estimated with Kaplan-Meier estimators. Blue dashed line helps to identify median PFS and OS.

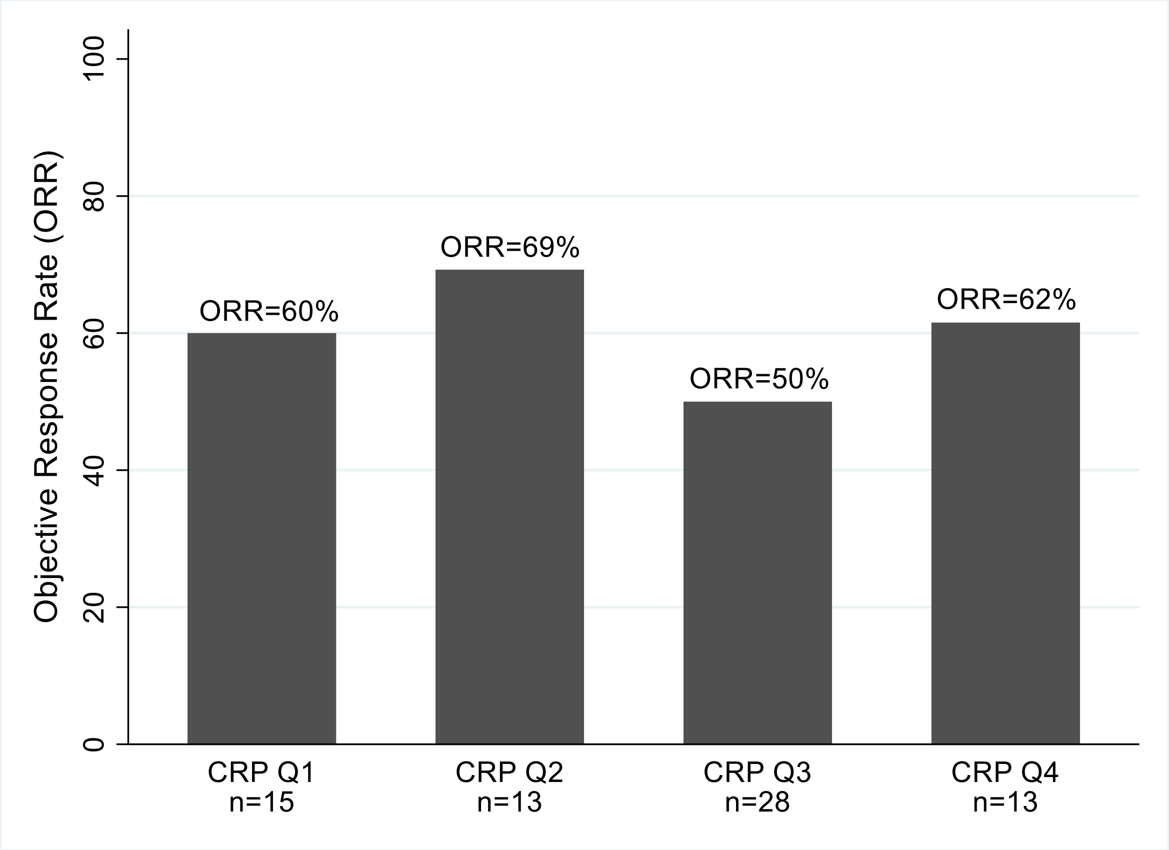

Figure S2. External validation of baseline CRP as a predictor of ICI therapy objective response rate in the Nuremberg cohort ($n = 69$). Data are only for patients with observed baseline CRP and a response assessment other than NE (not evaluated). CRP quartile cut-offs were taken from the Graz cohort. Thus, numbers of patients in the CRP quartiles are not balanced. CRP quartile cut-offs were as follows: Q1: CRP ≤ 7.7 mg/L, Q2: CRP > 7.7mg/L but ≤ 21.6 mg/L, Q3: CRP > 21.6 mg/L but ≤ 66.1 mg/L, and Q4: CRP > 66.1 mg/L. Abbreviations: ORR—Objective response rate, CRP—C-reactive protein, Q—Quartile.

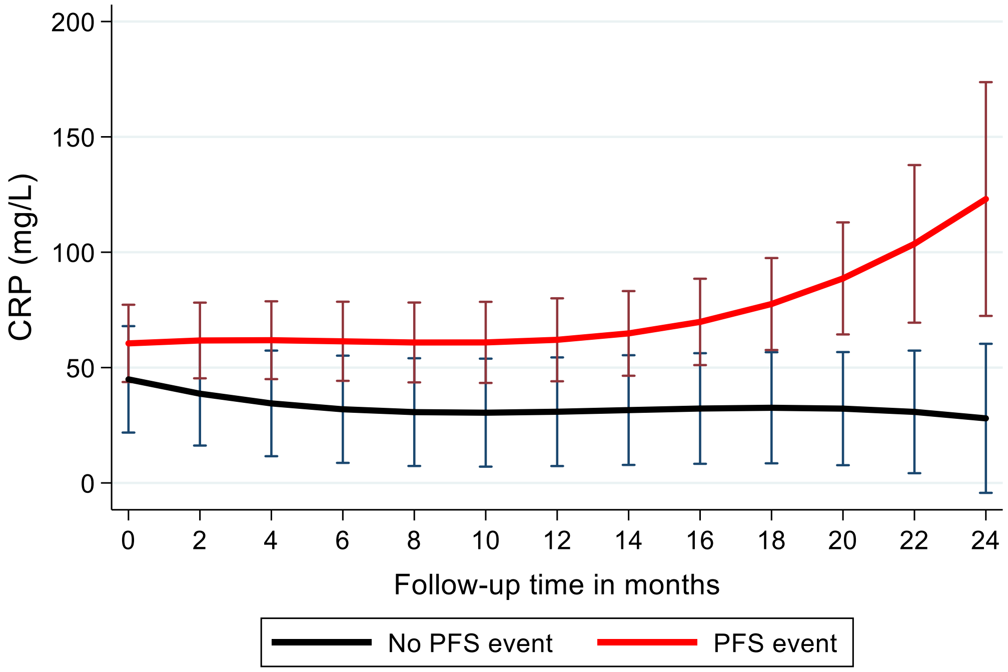

Figure S3. Margins plot of average CRP trajectories in patients who did (red line) and did not (black line) develop a PFS event during follow-up. Data are from a mixed model allowing for interactions between PFS status and linear, quadratic, and cubic follow-up time. This analysis does not account for (potentially informative) censoring. Range bars represent 95% confidence bands. Abbreviations: CRP—C-reactive protein, PFS—Progression-free survival.

Table 1. ICI therapy response categories in the Graz and Nuremberg cohort. Data are absolute counts (%). The response categories were assessed by treating physicians in analogy to immune-related response evaluation criteria in solid tumors (irRECIST) but do not constitute formal radiographic responses assessed by an independent trial radiologist. Most patients who were not evaluated (NE) had disease progression / died before radiographic response assessment.

**Physician-assessed radiographic response category**	**Graz: n (%)**	**Nuremberg: n (%)**
Complete remission (CR)	2 (2%)	0 (0%)
Partial remission (PR)	16 (18%)	40 (40%)
Stable disease (SD)	25 (28%)	11 (11%)
Progressive disease (PD)	31 (34%)	18 (18%)
Not evaluated (NE)	16 (18%)	32 (32%)

Table S2. Baseline characteristics in the Graz cohort in patients with available baseline CRP (n = 85)—Distribution overall and by CRP level. For this tabulation, CRP was dichotomized into a binary variable using an empirical cut-off at the 75th percentile of its distribution (Q3, 66.1 mg/L). Data are medians (25th–75th percentile) for continuous data, and absolute frequencies (%) for count data. n (%miss.) reports the number of patients with fully observed data for the respective variable (% missing). * p-values are from rank-sum tests, Fisher's exact tests, and χ^2-tests, as appropriate. ** Variables in the section "Treatment prior ICI" are with n = 32 patients, and the missingness percentage was consequently scaled to 100% for n = 32. Abbreviations: CRP—C-reactive protein, ICI—Immune checkpoint inhibitor, BMI—Body mass index, ECOG—Eastern Cooperation Oncology Group performance status, NSCLC—Non-small cell lung cancer, EGFR—Epidermal growth factor receptor, EML4-ALK—Echinoderm microtubule associated protein-like 4 anaplastic lymphoma kinase, ROS1—ROS proto-oncogene 1, BRAF—v-Raf murine sarcoma viral oncogene homolog B, PD-L1—programmed death ligand 1, RTx—Radiotherapy, CTx—Chemotherapy, RCTx—Chemoradiation.

**Variable**	**n (% miss.)**	**Overall (n = 85)**	**Baseline CRP ≤66.1 mg/L (i.e. Q3, n = 4)**	**Baseline CRP >66.1 mg/L (i.e. Q3, n = 21)**	**p***
**Demographic characteristics**					
Age at ICI initiation (years)	85 (0%)	67 (59–74)	68 (60–74)	61 (52–75)	0.196
Female Gender	85 (0%)	40 (47%)	32 (50%)	8 (38%)	0.343
BMI at ICI initiation (kg/m^2)	80 (6%)	24.2 (20.9–27.5)	24.2 (21.0–27.3)	25.2 (20.7–27.8)	0.892
Charleson comorbidity index at ICI initiation (points)	85 (0%)	8 (5–9)	8 (5–9)	7 (6–9)	0.992
Past or present smoker	82 (4%)	64 (78%)	48 (79%)	16 (76%)	0.811
ECOG at ICI initiation (points)	57 (33%)	0 (0–1)	0 (0–1)	1 (0–1)	0.555
Second primary malignancy at any time	81 (5%)	18 (22%)	18 (30%)	0 (0%)	**0.004**
**Tumor variables**					
Adenocarcinoma	85 (0%)	60 (71%)	48 (75%)	12 (57%)	0.119
Stage IV at initial NSCLC diagnosis	85 (0%)	52 (61%)	36 (56%)	16 (76%)	0.104
EGFR mutation	69 (19%)	2 (3%)	1 (2%)	1 (6%)	0.435

EML4-ALK rearrangement	69 (19%)	1 (1%)	1 (2%)	0 (0%)	0.999
ROS1 overexpression	57 (33%)	0 (0%)	0 (0%)	0 (0%)	N/A
BRAF mutation	21 (75%)	2 (10%)	1 (7%)	1 (14%)	0.999
PD-L1 expression (%)	65 (24%)	40 (1–80)	50 (1–80)	20 (1–80)	0.662
**Treatment prior ICI**					
Primary treatment intent: curative**	85 (0%)	30 (35%)	25 (39%)	5 (24%)	0.204
---Any neoadjuvant therapy (RTx, CTx, RCTx)	30 (0%)	8 (27%)	7 (28%)	1 (20%)	0.712
---Any definitive RCTx	30 (0%)	6 (20%)	5 (20%)	1 (20%)	0.999
---Any curative surgery	30 (0%)	21 (70%)	17 (68%)	4 (80%)	0.999
---Any adjuvant therapy (CTx, RTx)	30 (0%)	11 (37%)	7 (28%)	4 (80%)	**0.047**
**ICI treatment variables**					
ICI treatment line	85 (0%)	/	/	/	0.812
---1st-line	/	35 (41%)	25 (39%)	10 (48%)	/
---2nd-line	/	42 (49%)	33 (52%)	9 (43%)	/
---3rd, 4th, or 5th-line	/	8 (9%)	6 (9%)	2 (10%)	/
ICI agent	85 (0%)	/	/	/	0.198
---Nivolumab	/	48 (56%)	38 (59%)	10 (48%)	/
---Pembrolizumab	/	34 (40%)	25 (39%)	9 (43%)	/
---Atezolizumab	/	3 (3%)	1 (2%)	2 (10%)	/
Number of ICI cycles	76 (11%)	5 (2–15)	7 (4–19)	2 (1–3)	**<0.0001**

Table 3. Multivariable models for objective response rate, progression-free and overall survival in the Graz cohort. Data are from multivariable Cox proportional hazards regression models. Variables for multivariable adjustment were selected based on a significant univariable association with the outcome (Table 2). Baseline CRP is specified as a log2-transformed variable. Abbreviations: PFS—Progression-free survival, OS—Overall survival, HR—Hazard ratio, 95%CI—95% confidence interval (Wald test p-value), CRP—C-reactive protein, ICI—Immune-checkpoint inhibitor, N/A—Not applicable, Ref.—Reference category.

	**Multivariable Model #1: ORR**		**Multivariable Model #2: PFS**		**Multivariable Model #3: OS**	
Variable	Adjusted HR	95%CI (p)	Adjusted HR	95%CI (p)	Adjusted HR	95%CI (p)
Baseline CRP (per doubling)	0.66	0.47–0.91 ($p = 0.011$)	1.37	1.16–1.63 ($p < 0.0001$)	1.42	1.18–1.71 ($p < 0.0001$)
Age (per 10 years increase)	2.45	1.11–5.40 ($p = 0.026$)	0.78	0.63–0.95 ($p = 0.014$)	0.69	0.55–0.87 ($p = 0.002$)
Stage IV at initial diagnosis	N/A	N/A	1.65	0.92–2.97 ($p = 0.094$)	1.56	0.83–2.96 ($p = 0.170$)
Female Gender	N/A	N/A	N/A	N/A	0.45	0.25–0.80 ($p = 0.007$)
Line of ICI therapy: ---1st-line	N/A	N/A	N/A	N/A	Ref.	Ref.
---2nd-line	N/A	N/A	N/A	N/A	2.37	1.20–4.66 ($p = 0.013$)
---3rd or later line	N/A	N/A	N/A	N/A	2.35	0.87–6.36

(p=0.093)

Table 4. Multivariable model for objective response rate, progression-free and overall survival in the Graz cohort adjusted for NLR, LDH and LIPI Score. Data are from multivariable Cox proportional hazards regression models. Abbreviations: PFS—Progression-free survival, OS – Overall survival, HR – Hazard ratio, 95%CI—95% confidence interval (Wald test *p*-value), CRP—C-reactive protein, NLR—Neutrophil/Lymphocyte ratio, LDH—lactat dehydrogenase, ICI—Immune-checkpoint inhibitor, N/A—Not applicable, Ref.—Reference category.

Multi-variable model	Variable	ORR—Odds Ratio (95%CI, *p*)	PFS—Hazard ratio (95%CI, *p*)	OS—Hazard ratio (95%, *p*)
#1	CRP (per doubling)	0.71 (0.51–0.98, $p = 0.036$)	1.40 (1.17–1.68, $p < 0.0001$)	1.28 (1.07–1.53, $p = 0.006$)
	NLR (per doubling)	1.00 (0.54–1.83, $p = 0.992$)	1.14 (0.82–1.59, $p = 0.444$)	1.30 (0.94–1.80)
#2	CRP (per doubling)	0.64 (0.47–0.89, $p = 0.008$)	1.48 (1.23–1.77, $p < 0.0001$)	1.40 (1.18–1.68, $p < 0.0001$)
	LDH (per doubling)	1.01 (0.40–2.55, $p = 0.975$)	1.19 (0.81–1.76, $p = 0.373$)	1.20 (0.81–1.76, $p = 0.368$)
#3	CRP (per doubling)	0.71 (0.51–0.99, $p = 0.041$)	1.44 (1.20–1.73, $p < 0.0001$)	1.31 (1.10–1.57, $p = 0.003$)
	LIPI: 0 points	Ref.	Ref.	Ref.
	---1 point	1.64 (0.42–6.44, $p = 0.476$)	1.10 (0.56–2.16, $p = 0.780$)	1.18 (0.57–2.46, $p = 0.659$)
	---2 points	0.73 (0.11–4.97, $p = 0.744$)	1.66 (0.76–3.62, $p = 0.202$)	2.20 (0.95–5.11, $p = 0.067$)

Table 5. Comparison of the baseline characteristics of the Graz and Nuremberg cohort. Data are for patients with observed baseline CRP ($n = 85$ and $n = 101$). Some data were not available (n/a) in the Nuremberg cohort. Summary measures are medians (25th–75th percentile) for continuous data, and absolute frequencies (%) for count data. n (%miss.) reports the number of patients with fully observed data for the respective variable (% missing). *ECOG data in the Nuremberg cohort were not exclusively from ICI initiation but from initiation of 1st-line therapy. **Variables in the section "Treatment prior ICI" are with $n = 30$ patients, and the missingness percentage was consequently scaled to 100% for $n = 30$. Abbreviations: CRP—C-reactive protein, ICI—Immune checkpoint inhibitor, n/a – data not available in the Nuremberg cohort, BMI—Body mass index, ECOG—Eastern Cooperation Oncology Group performance status, NSCLC—Non-small cell lung cancer, EGFR—Epidermal growth factor receptor, EML4-ALK—Echinoderm microtubule associated protein-like 4 anaplastic lymphoma kinase, ROS1—ROS proto-oncogene 1, BRAF—v-Raf murine sarcoma viral oncogene homolog B, PD-L1—programmed death ligand 1, RTx—Radiotherapy, CTx—Chemotherapy, RCTx—Chemoradiation.

	Graz cohort with baseline CRP observed (*n* = 85)		Nuremberg cohort with baseline CRP observed (*n* = 101)	
**Variable**	**n (% miss.)**	**Summary measure (25th–75th percentile, or %)**	**n (% miss.)**	**Summary measure (25th–75th percentile, or %)**
**Demographic characteristics**				
Age at ICI initiation (years)	85 (0%)	67 (59–74)	101 (0%)	68 (60–74)
Female Gender	85 (0%)	40 (47%)	101 (0%)	34 (34%)
BMI at ICI initiation (kg/m^2)	80 (6%)	24.2 (20.9–27.5)	0 (100%)	n/a

Charleson comorbidity index at ICI initiation (points)	85 (0%)	8 (5–9)	0 (100%)	n/a
Past or present smoker	82 (4%)	64 (78%)	0 (100%)	n/a
ECOG at ICI initiation (points)*	57 (33%)	0 (0–1)	86 (15%)	0 (0–1)
Second primary malignancy at any time	81 (5%)	18 (22%)	0 (100%)	n/a
**Tumor variables**				
Adenocarcinoma	85 (0%)	60 (71%)	101 (0%)	59 (58%)
Stage IV at initial NSCLC diagnosis	85 (0%)	52 (61%)	101 (0%)	64 (64%)
EGFR mutation	69 (19%)	2 (3%)	60 (40%)	2 (4%)
EML4-ALK rearrangement	69 (19%)	1 (1%)	60 (40%)	0 (0%)
ROS1 overexpression	57 (33%)	0 (0%)	60 (40%)	0 (0%)
BRAF mutation	21 (75%)	2 (10%)	60 (40%)	1 (2%)
PD-L1 expression (%)	65 (24%)	40 (1–80)	56 (45%)	60 (60–90)
**Treatment prior ICI**				
Primary treatment intent: curative**	85 (0%)	30 (35%)	101 (0%)	37 (37%)
---Any neoadjuvant therapy (RTx, CTx, RCTx)	30 (0%)	8 (27%)	37 (0%)	3 (8%)
---Any definitive RCTx	30 (0%)	6 (20%)	0 (0%)	0 (0%)
---Any curative surgery	30 (0%)	21 (70%)	0 (0%)	18 (49%)
---Any adjuvant therapy (CTx, RTx)	30 (0%)	11 (37%)	0 (0%)	4 (11%)
**ICI treatment variables**				
ICI treatment line	85 (0%)	/	101 (0%)	/
---1st-line	/	35 (41%)	/	45 (45%)
---2nd-line	/	42 (49%)	/	41 (41%)
---3rd, 4th, or 5th-line	/	8 (9%)	/	15 (15%)
ICI agent	85 (0%)	/	101 (0%)	/
---Nivolumab	/	48 (56%)	/	55 (54%)
---Pembrolizumab	/	34 (40%)	/	46 (46%)
---Atezolizumab	/	3 (3%)	/	0 (0%)
ICI in more than 1 treatment line	85 (0%)	1 (1%)	101 (0%)	0 (0%)
Number of ICI cycles	76 (11%)	5 (2–15)	0 (100%)	7 (3–14)
**Laboratory variables**				
CRP at baseline (mg/L)	85 (6%)	21.6 (7.7–66.1)	101 (0%)	32 (11–64)

STROBE Statement—checklist of items that should be included in reports of observational studies.

	Item No	Recommendation	Reported on page
**Title and abstract**	1	(*a*) Indicate the study's design with a commonly used term in the title or the abstract	1,4
		(*b*) Provide in the abstract an informative and balanced summary of what was done and what was found	4–5
		**Introduction**	
Background/rationale	2	Explain the scientific background and rationale for the investigation being reported	6–7
Objectives	3	State specific objectives, including any prespecified hypotheses	7
		**Methods**	
Study design	4	Present key elements of study design early in the paper	8,9
Setting	5	Describe the setting, locations, and relevant dates, including periods of recruitment, exposure, follow-up, and data collection	8,9
Participants	6	(*a*) *Cohort study*—Give the eligibility criteria, and the sources and methods of selection of participants. Describe methods of follow-up *Case-control study*—Give the eligibility criteria, and the sources and methods of case ascertainment and control selection. Give the rationale for the choice of cases and controls *Cross-sectional study*—Give the eligibility criteria, and the sources and methods of selection of participants	8,9
		(*b*) *Cohort study*—For matched studies, give matching criteria and number of exposed and unexposed *Case-control study*—For matched studies, give matching criteria and the number of controls per case	N/A
Variables	7	Clearly define all outcomes, exposures, predictors, potential confounders, and effect modifiers. Give diagnostic criteria, if applicable	8,9
Data sources/ measurement	8*	For each variable of interest, give sources of data and details of methods of assessment (measurement). Describe comparability of assessment methods if there is more than one group	8–11
Bias	9	Describe any efforts to address potential sources of bias	8–11
Study size	10	Explain how the study size was arrived at	8,9
Quantitative variables	11	Explain how quantitative variables were handled in the analyses. If applicable, describe which groupings were chosen and why	10–11
Statistical methods	12	(*a*) Describe all statistical methods, including those used to control for confounding	10–11
		(*b*) Describe any methods used to examine subgroups and interactions	10–11
		(*c*) Explain how missing data were addressed	10,11,28,29
		(*d*) *Cohort study*—If applicable, explain how loss to follow-up was addressed *Case-control study*—If applicable, explain how matching of cases and controls was addressed *Cross-sectional study*—If applicable, describe analytical methods taking account of sampling strategy	10–11
		(*e*) Describe any sensitivity analyses	10–11
		**Results**	
Participants	13*	(a) Report numbers of individuals at each stage of study—eg numbers potentially eligible, examined for eligibility, confirmed eligible, included in the study, completing follow-up, and analysed	12–16
		(b) Give reasons for non-participation at each stage	N/A
		(c) Consider use of a flow diagram	N/A

Descriptive data	14*	(a) Give characteristics of study participants (eg demographic, clinical, social) and information on exposures and potential confounders	12–16, 28–31
		(b) Indicate number of participants with missing data for each variable of interest	38–31
		(c) *Cohort study*—Summarise follow-up time (eg, average and total amount)	12
Outcome data	15*	*Cohort study*—Report numbers of outcome events or summary measures over time	12
		Case-control study—Report numbers in each exposure category, or summary measures of exposure	*N/A*
		Cross-sectional study—Report numbers of outcome events or summary measures	*N/A*
Main results	16	(*a*) Give unadjusted estimates and, if applicable, confounder-adjusted estimates and their precision (eg, 95% confidence interval). Make clear which confounders were adjusted for and why they were included	13–16,30,32,
		(*b*) Report category boundaries when continuous variables were categorized	12
		(*c*) If relevant, consider translating estimates of relative risk into absolute risk for a meaningful time period	16,32
Other analyses	17	Report other analyses done—eg analyses of subgroups and interactions, and sensitivity analyses	16
**Discussion**			
Key results	18	Summarise key results with reference to study objectives	17
Limitations	19	Discuss limitations of the study, taking into account sources of potential bias or imprecision. Discuss both direction and magnitude of any potential bias	20
Interpretation	20	Give a cautious overall interpretation of results considering objectives, limitations, multiplicity of analyses, results from similar studies, and other relevant evidence	17–20
Generalisability	21	Discuss the generalisability (external validity) of the study results	21
**Other information**			
Funding	22	Give the source of funding and the role of the funders for the present study and, if applicable, for the original study on which the present article is based	23

*Give information separately for cases and controls in case-control studies and, if applicable, for exposed and unexposed groups in cohort and cross-sectional studies. **Note:** An Explanation and Elaboration article discusses each checklist item and gives methodological background and published examples of transparent reporting. The STROBE checklist is best used in conjunction with this article (freely available on the Web sites of PLoS Medicine at http://www.plosmedicine.org/, Annals of Internal Medicine at http://www.annals.org/, and Epidemiology at http://www.epidem.com/). Information on the STROBE Initiative is available at www.strobe-statement.org.

Von Elm. E.; Altman, D.G.; Egger, M.; Pocock, S.J.; Gøtzsche, P.C.; Vandenbroucke, J.P.; STROBE Initiative. The Strengthening the Reporting of Observational Studies in Epidemiology (STROBE) statement: guidelines for reporting observational studies. *Lancet* **2007**, *370*, 1453–1457
